# Are animal models predictive for human postmortem muscle protein degradation?

**DOI:** 10.1007/s00414-017-1643-1

**Published:** 2017-07-18

**Authors:** Bianca Ehrenfellner, Angela Zissler, Peter Steinbacher, Fabio C. Monticelli, Stefan Pittner

**Affiliations:** 1 Department of Cell Biology and Physiology, University of Salzburg, Hellbrunnerstr. 34, 5020 Salzburg, Austria; 2 Department of Forensic Medicine and Forensic Neuropsychiatry, University of Salzburg, Ignaz-Harrer-Straße 79, 5020 Salzburg, Austria

**Keywords:** Postmortem interval (PMI), Skeletal muscle, Protein, Degradation, Human, Animal model

## Abstract

A most precise determination of the postmortem interval (PMI) is a crucial aspect in forensic casework. Although there are diverse approaches available to date, the high heterogeneity of cases together with the respective postmortal changes often limit the validity and sufficiency of many methods. Recently, a novel approach for time since death estimation by the analysis of postmortal changes of muscle proteins was proposed. It is however necessary to improve the reliability and accuracy, especially by analysis of possible influencing factors on protein degradation. This is ideally investigated on standardized animal models that, however, require legitimization by a comparison of human and animal tissue, and in this specific case of protein degradation profiles. Only if protein degradation events occur in comparable fashion within different species, respective findings can sufficiently be transferred from the animal model to application in humans. Therefor samples from two frequently used animal models (mouse and pig), as well as forensic cases with representative protein profiles of highly differing PMIs were analyzed. Despite physical and physiological differences between species, western blot analysis revealed similar patterns in most of the investigated proteins. Even most degradation events occurred in comparable fashion. In some other aspects, however, human and animal profiles depicted distinct differences. The results of this experimental series clearly indicate the huge importance of comparative studies, whenever animal models are considered. Although animal models could be shown to reflect the basic principles of protein degradation processes in humans, we also gained insight in the difficulties and limitations of the applicability of the developed methodology in different mammalian species regarding protein specificity and methodic functionality.

## Introduction

Determination of the postmortem interval (PMI) is one of the most challenging and difficult tasks in daily forensic casework due to limitations of accurate and reliable methods. Precise estimation of the time of death is crucial for criminal law as it often validates a witness’s statement, possibly limits the number of suspects and assesses alibies. Especially in cases with multiple dead bodies within temporal proximity, information about the succession of events is often of utmost importance. Although there is a lot of research focused on new attempts for PMI estimation and on the improvement of existing approaches [1–6], novel methods for time of death estimation that comply with the requirements of practice are still of high demand. In early decomposition stages, first physical changes, effects of cellular breakdown, autolytic activity and structural alterations in tissue can be detected [7]. In this postmortem period characteristic changes such as *rigor mortis* (stiffening and relaxation of skeletal muscle), *livor mortis* (accumulation of blood and blood products at lower parts of the body), *algor mortis* (changes in body core temperature) as well as supravital reactions [8, 9], such as response to electrical or pharmacological excitation [10] are applied for the estimation of the time since death in every day forensic work. These parameters, however, are highly dependent upon individual and environmental factors, such as temperature, humidity, age, body size, cause of death, or pathological precondition [11, 12] and are generally restricted to early postmortem time periods (i.e., the first 36 h postmortem, hpm) [11]. PMI estimation in later stages is usually even more difficult as the range of available methods is far smaller. In postmortem periods, starting from approximately 72 hpm, forensic entomology, the analyses of developmental stages of cadaver-feeding insects, can sometimes provide information about the minimum PMI [13]. But also, this method is limited in many cases, e.g., by insect accessibility (bodies discovered indoor, drowned, or covered bodies, etc...), or certain threshold temperatures for the development of different species [12, 14]. PMI estimation in even later stages postmortem, such as in mummified corpses is even more difficult. This is only rarely possible solely on behalf of biomedical data such as autopsy findings [15], but is most often limited to other sources of information, like evidence found at the scene (call register, transport tickets or newspaper), testimonies or confessions. These data are however, highly case dependent and cannot be transferred from one to another. New biomedical approaches to account for cases like these, and thus additional research in this field is highly required.

An important consideration is to achieve highest possible precision, but reliability, cost, and time effort as well as handling play a central role in the establishment of new methods for forensic routine work and often prevent successful application [16]. Recently, an approach based on skeletal muscle protein degradation was reported for PMI estimation. Various alterations over a postmortem period of 10 days were observed in an animal model [2] and in further consequence, verified in human forensic cases [17]. Comparable changes were obtained in humans that significantly correlated with time and temperature [17]. A first successful application of this approach already proved the viability in a comparison of two cases, in which major influencing factors (e.g., temperature) could be excluded [18]. In general, samples obtained in daily forensic practice are difficult to compare to each other due to varying individual influencing factors (e.g., age, BMI, cause of death, etc.) and variable conditions within the PMI (temperature, humidity, etc...). To describe the basic principles of biological processes and certain influencing factors, animal models are often inevitable [19–22]. However, obtained results cannot be applied to human cases without further considerations and appropriately comparative studies, which represent a very common task, in bridging basic and applied research. Especially in forensic science, pigs are commonly used as model organisms due to their comparable body size and physiology to humans [23, 24], but require large scale experimental setups and according facilities. Rodents, on the other hand, are cheap and easy in availability and handling, but provide fewer sample material. To evaluate the legitimacy of both of these models, and to eventually benefit from the advantages of both, we conducted an experimental series, comparing protein degradation events in humans, pigs, and mice. We aimed to identify qualitative alterations in three different species as well as to compare eventual differences and similarities and thus validate the legitimacy for further research on the respective animal model. Additionally we expanded the set of possible target proteins to detect further degradation patterns and thereby identify additional viable postmortem interval markers.

## Material and methods

As a standard for comparison we selected three human cases from our database with highly differing PMI to be able to illustrate the diversity of possible protein band patterns. Since there were no native samples available we selected a case with a small PMI (~0.5 days postmortem, dpm), to represent early postmortem phases. This sample was taken from a 76-year-old female, who died on respiratory failure in a hospital. Intermediate PMIs are represented by the case of an 82-year-old female who also died on respiratory failure and was found few days later (~2.5 dpm) in her home. The third sample was taken from an already putrid body of an 81-year-old man found outside. Application of any of the available methods for time of death estimation was impossible in this case. Solely forensic entomology and non-biomedical evidence, gathered from police investigations resulted in a rough estimate of ~40.0 dpm. Notably, protein degradation analyses still revealed characteristic protein profiles.

These cases were compared to porcine samples from a previous study. Here, the earliest (0 dpm), an intermediate (2.5 dpm) and the latest PMI available (10.0 dpm) were selected. Additionally, we obtained postmortem muscle samples from mice as a pilot approach to evaluate the legitimacy of this frequently used model organism. In order to account for possible earlier protein decomposition events, as they can be expected when comparing literature data [25], we processed 0, 0.8, and 1.5 dpm muscle samples according to our standard protocols [17] (Table 1).

**Table 1:** Overview of the samples used for this comparative study. Short, intermediate, and long postmortem intervals (PMI) in humans, pigs, and mice were defined and selected, and compared on behalf of qualitative postmortem protein degradation events. Ambient temperature (*T*_amb_) is depicted in °C, PMI in days postmortem (dpm)

	*T*_amb_ [°C]	Sample 1 short PMI [dpm]	Sample 2 intermediate PMI [dpm]	Sample 3 long PMI [dpm]
Human	15–20	~0.5	~2.5	~40.0
Pig	21	0	2.5	10.0
Mouse	20	0	0.8	1.5

Prior to analyses, small samples (~5 × 5 × 5 mm) were removed from thigh muscles of each species and snap frozen in liquid nitrogen. Homogenization was performed by cryogenic grinding and sonication. Protein concentrations were measured using Pierce BCA Assay Kit. SDS-PAGE and western blotting analyses were performed according to standard protocols [17] with slight modifications. The amount of total protein was individually adapted for each species to obtain comparable band intensities. The following primary antisera were used: mouse monoclonal anti-tropomyosin, mouse monoclonal anti-alpha actinin, mouse monoclonal anti-alpha tubulin, mouse monoclonal anti-fast skeletal muscle troponin T, mouse monoclonal anti-vinculin, mouse monoclonal anti-desmin, mouse monoclonal anticardiac troponin T, and mouse monoclonal anti-vimentin. HRP-conjugated polyclonal goat anti-mouse was applied as secondary antibody. For detection, a chemiluminescent substrate (Roti-Lumin plus) was applied and the signal was captured by a digital chemiluminescence detector. Protein bands were quantified by ImageJ software (v.1.48 NIH, National Institutes of Health, USA). All signals <1% of the respective dominant band were considered background (i.e., not regarded a protein band). Band patterns obtained from the fresh samples (with respective caution in the human case) were considered the native form of the protein. Additional or disappearing band signals in samples with higher PMI were regarded a degradation event.

## Results

As expected, no major differences in sample preparation and handling were observed, apart from sample dilution to obtain equal band intensities. The protocols can be applied for any of the tested species, which enabled sensible comparison of protein degradation profiles.

**Fig. 1:**
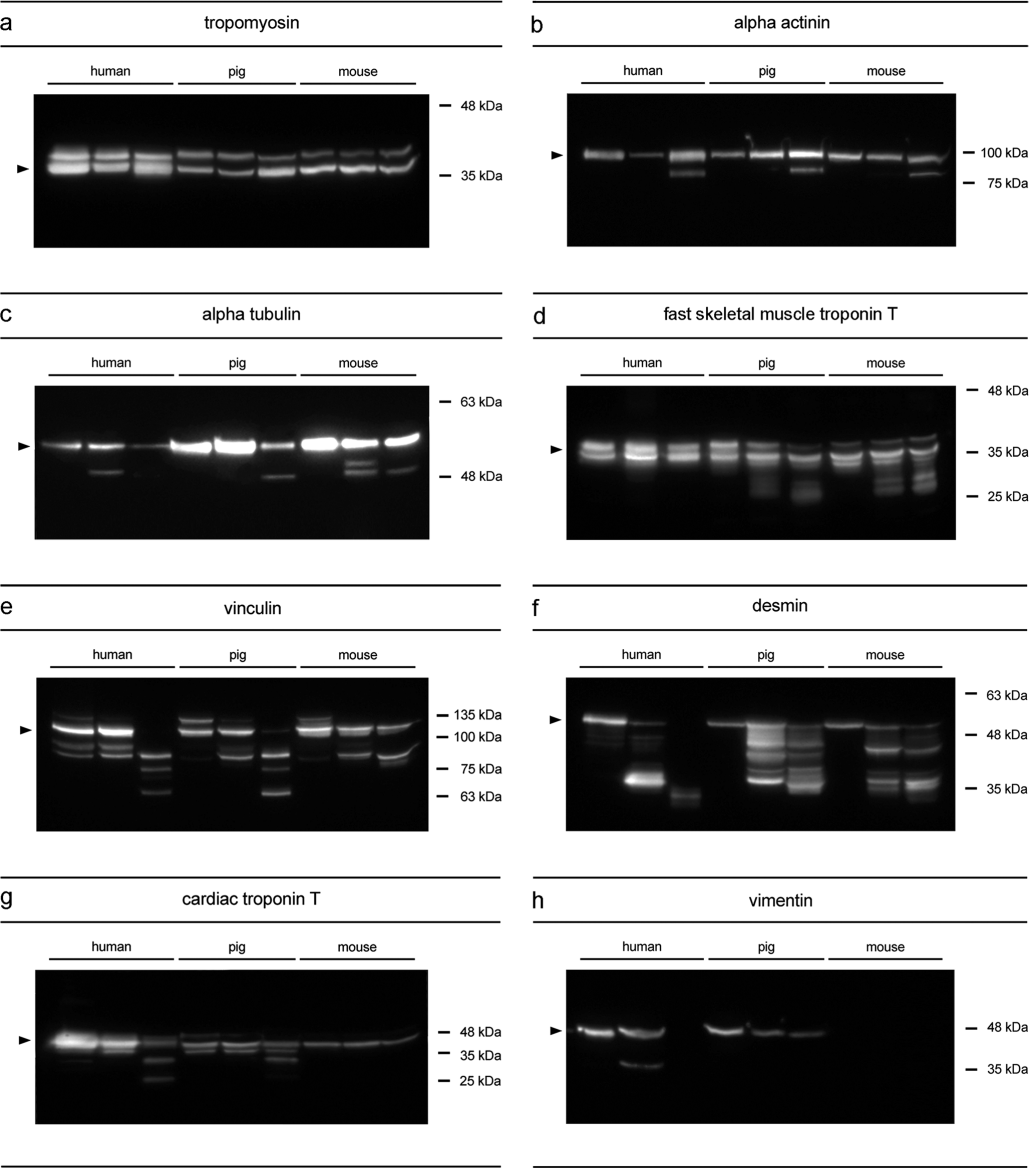
Western blot analyses of postmortem protein degradation in three different species. Muscle samples from small, intermediate and advanced PMI were immuno-labeled using antisera against tropomyosin (**a**), alpha actinin (**b**), alpha tubulin (**c**), fast skeletal muscle troponin T (**d**), vinculin (**e**), desmin (**f**), cardiac troponin T (**g**), and vimentin (**h**). The loss of a native band, as well as appearance of an additional band compared to the early phase was considered a degradation event. Except for **a** such events occurred in every protein analyzed. Most of the degradation events were detected in comparable fashion in all three of the investigated species, such as the appearance of a degradation product in **b**, **c**, **e**, and **f**. Some others, however, only occurred in some species, such as the degradation products in **d** in pigs and mice, in **g** in humans and pigs or **h** solely in humans. The loss of a native band or degradation products was detected in **f** and **h** in humans, as well as **e** in humans and pigs. *Black arrow heads* indicate the native band of the respective protein

Degradation analysis of tropomyosin resulted in two bands that appeared consistently throughout all tested species (Fig. 1a). These bands represent two isoforms with molecular weights of approximately 36 and 38 kDa. This stable state retained without the loss of native bands or appearance of any degradation products.

Native alpha actinin (~100 kDa) also remained stable in all species during the investigated courses of postmortem protein degradation (Fig. 1b). Samples representing larger PMIs exhibited an additional protein band of similar molecular weight (~ 80 kDa) within all three species.

Similarly, the native band of alpha tubulin (~55 kDa) was continuously detectable, however, with very weak intensity in the human sample with the highest PMI within the dataset (Fig. 1c). Early phases after death are apparently characterized by a single native band (~55 kDa), whereas, subsequently a degradation product of approximately 50 kDa, appeared in humans and both animal models. Notably, this degradation product was not detectable in the latest human sample. An additional degradation product at ~52 kDa emerged in the intermediate murine sample.

Analysis of fast skeletal muscle troponin T revealed a native double band in all tested species between approximately 40 kDa and 35 kDa (Fig. 1d). The initial state of murine samples already comprised an additional protein band (~33 kDa) that was also detected in the porcine degradation progress. Additionally, similar degradation events with several appearing protein bands between ~33 and ~25 kDa were observed in comparable fashion in both of the animal models. However, no changes from the initial pattern were observed in human samples.

Analyses of vinculin resulted in an intact band at ~117 kDa as well as a protein band with slightly higher molecular weight, commonly suggested as a splice variant [26] in the earliest PMIs in all species (Fig. 1e). The splice variant band vanished with increasing PMI in all tested species. Additionally, two protein bands at ~90 and ~84 kDa were observed in the human sample with the shortest PMI. The larger one appeared to be species specific, whereas the lower one was also detected as a degradation product in mice and pigs. Larger PMIs revealed similar degradation events in humans and pigs with degradation products between 75 and 63 kDa. Mouse samples depicted a different pattern with a protein band detected at ~78 kDa.

Native desmin was detected as a single band at ~50 kDa in all three species (Fig. 1f). Degradation behavior is comprised by a large number of protein bands and was difficult to discriminate. However, individual degradation progresses can be described due to characteristic patterns. In the initial state, a single native band (~50 kDa) is clearly detectable in the animal models, whereas in humans several additional smaller bands appeared between ~50 and ~45 kDa. As the PMI increases, the number of protein bands increased in the range between ~50 and 38 kDa in all three species. In humans, additional degradation products (<35 kDa) appeared in further consequence, whereas the native band and other degradation products disappeared.

A native cardiac troponin T band was detected in all examined samples with a molecular weight of approximately 43 kDa (Fig. 1g). The initial state in porcine muscle comprised a second protein band (~38 kDa) that persisted over the analyzed period. The early human sample appeared rather inconclusive concerning the native state. However, a band signal similar to that in porcine samples was detectable during the subsequent degradation process. Another comparable degradation event (appearance of a degradation product at ~33 kDa) was observed in human and porcine samples of the largest PMI. In addition, human samples depicted a third degradation product at ~25 kDa. Analysis of murine samples, however, did not reveal any degradation event compared to the native state during the investigated time period.

Analysis of vimentin revealed characteristic protein patterns in humans and pigs with a native band at ~58 kDa; however, no protein band at all in any of the tested mouse samples (Fig. 1h). A degradation product was observed in humans at ~48 kDa. Subsequently both, the native band and the degradation product were undetectable. Although there was no degradation product detectable in pigs, the native band distinctly decreased.

## Discussion

With this experimental series, we demonstrated important interspecies similarities in postmortem muscle protein degradation as well as certain distinct alterations of protein profiles on a qualitative basis that can be further investigated on a quantitative basis to examine PMI correlations. This is a mandatory aspect for the legitimacy of animal models in research on postmortem markers for PMI estimation. Furthermore, we were able to propose several additional viable biomarkers for the analysis of postmortem changes in both, the animal model and in humans.

Most of the proteins analyzed depicted very similar, almost identical, native states (in particular, early states in case of human samples), which is a strong indication for the validity of animals as model organism for humans. In some cases, degradation events occurred in almost identical fashion across different species. The loss of a native band in vinculin, or the appearance of degradation products of similar molecular weight in alpha actinin, alpha tubulin, and vinculin appear highly conserved across all species investigated. Very similar changes are also reported in other species such as chicken [27] or sheep [26]. Some others of the observed degradation events were only detected in mice, such as the appearance of a ~52 kDa degradation product in alpha tubulin and a ~85 kDa degradation product in vinculin. Whether this depicts species specifity or a transition state, which is simply not detected in the other species due to the low temporal resolution, remains to be determined. Similarly, some degradation events were only detected in a single human sample, the one with the highest PMI. The loss of a native band and of degradation products in desmin and vinculin as well as the appearance of a ~30 kDa degradation product in desmin and a ~30 kDa degradation product in cTnT, might as well be species specific, but are more likely induced by the much extended PMI compared to the other species. A long-term degradation study on the animal model could clarify this issue.

Vimentin, fTnT and cTnT western blots revealed distinct differences in pigs, mice, and humans. This highlights the general importance of comparative studies. Application of a specific antiserum for protein analysis in some species (such as vimentin in humans and pigs) does not necessarily apply for other species (such as using the same antiserum for mouse). This is a very important issue as, although there are several postmortem protein degradation events reported in various tissues, within animal models such as cattle [28], rats [29], or mice [25]; some of these alterations might be impossible to transfer to other species. Thus, when changes are described solely in the animal model they can eventually be completely irrelevant for human application. In the present study, a native band of cTnT was detected in all three species. However, there were no alterations of the protein profile in samples with higher PMI in mice, whereas distinct changes occurred in pigs and humans. Thus, analysis of cTnT degradation only in a mouse model would reveal no degradation event within a certain period of time which could in turn lead to wrong conclusions for humans (notably, these degradation events could occur in later PMIs in mice as well). Comparable behavior was illustrated in fTnT vice versa. While the native band was detected in all three species, alterations of protein profiles were only observed in pigs and mice, not in humans. Although fTnT western blots revealed promising results in both animal models, it is inadequate for application in humans within the investigated postmortem time period (however, degradation products might be detected in later PMIs).

Degradation processes, such as the loss of a native protein band or the appearance of degradation products have been previously described in many different species [2, 17, 25, 27, 30, 31]. However, to our knowledge this is the first single interspecies comparative study in this manner. Loss of native bands or appearance of degradation products occur due to autolysis [32, 33], which is a combination of tissue-specific metabolic processes that occur in many different species and are thus eventually comparable.

However, especially for the transfer of temporal information, species-specific differences have to be considered due to physical (e.g., body size, body temperature, composition of different tissues) and physiological (e.g., metabolic rate) differences, both very important factors in body decomposition [34]. Especially body size (even within a species) can be crucial for the rate of decomposition [35, 36] and by that might also contribute to distinct differences between species. Additional interspecies differences, such as reported for humans and pigs of comparable sizes [35] illustrate the complexity of influences on decomposition and make clear that temporal coherences between species should be generally avoided. Nevertheless, due to better comparability of physical influencing factors, future analysis of muscle protein degradation should also and preferably be conducted on human-sized animal models, whenever respective facilities are available.

Establishment of a method for time since death estimation requires coverage of two major aspects, the description of postmortem changes in standardized conditions and the analysis of influencing factors on the respective changes. Description of a degradation model from human forensic cases requires a very large data set and subsequent multifactorial cluster analysis. This is expensive and time consuming and difficult in the prediction of precision and reliability. Animal models, on the other hand, are successfully used in many fields of science and are regularly used in basic research to reflect humans in a standardized manner [19]. A controlled laboratory study on an animal model can provide all necessary conditions to investigate the basic principles of postmortem protein degradation processes and especially respective influencing factors. However, data transmission to the application in humans clearly requires verification in comparative studies.

To conclude, animal models are very important tools in biomedical research in general, and also specifically in forensic studies on PMI delimitation. Elaborated experimental setups under controlled conditions are essential to investigate the basic principles of postmortem alterations and their dependence on individual and environmental influencing factors. When sensibly considered, findings obtained from animal models can sufficiently aid to develop and adapt calculation models for PMI estimation in humans.

Analysis of postmortem muscle protein degradation is a viable tool for time since death estimation and in general, changes occur in comparable fashion in different species. However, some of the alterations appear species specific and can thus not be compared or even transferred at all. Comparative analyses of human and animal tissue are mandatory to legitimize research on animal models. Conclusions about temporal coherence of postmortem changes between different species are invalid within the presented experimental setup and are therefore refrained. Qualitative comparison and the possibility to examine influencing factors on a standard model, however, can enable significant improvements of mathematical models for the application in forensic routine work.
